# Therapeutic effects of rosemary (*Rosmarinus officinalis *L.) and its active constituents on nervous system disorders

**DOI:** 10.22038/ijbms.2020.45269.10541

**Published:** 2020-09

**Authors:** Mahboobeh Ghasemzadeh Rahbardar, Hossein Hosseinzadeh

**Affiliations:** 1 Pharmaceutical Research Center, Pharmaceutical Technology Institute, Mashhad University of Medical Sciences, Mashhad, Iran; 2 Department of Pharmacodynamics and Toxicology, School of Pharmacy, Mashhad University of Medical Sciences, Mashhad, Iran

**Keywords:** Addiction, Anticonvulsant, Antinociceptive, Neurodegenerative disease, Nervous system, Neuroprotective, Rosmarinus officinalis

## Abstract

Rosemary (*Rosmarinus officinalis* L.) is an evergreen bushy shrub which grows along the Mediterranean Sea, and sub-Himalayan areas. In folk medicine, it has been used as an antispasmodic, mild analgesic, to cure intercostal neuralgia, headaches, migraine, insomnia emotional upset, and depression. Different investigations have highlighted rosemary neuropharmacological properties as their main topics. Rosemary has significant antimicrobial, anti-inflammatory, anti-oxidant, anti-apoptotic, anti-tumorigenic, antinociceptive, and neuroprotective properties. Furthermore, it shows important clinical effects on mood, learning, memory, pain, anxiety, and sleep. The aim of the current work is to review the potential neuropharmacological effects of different rosemary extracts and its active constituents on nervous system disorders, their relevant mechanisms and its preclinical application to recall the therapeutic potential of this herb and more directions of future research projects. The data were gathered by searching the English articles in PubMed, Scopus, Google Scholar, and Web of Science. The keywords used as search terms were ‘*Rosmarinus officinalis*’, ‘rosemary’, ‘nervous system’, ‘depression’, ‘memory’, ‘Alzheimer’s disease’ ‘epilepsy’, ‘addiction’, ‘neuropathic pain’, and ‘disorders’. All kinds of related articles, abstracts and books were included. No time limitation was considered. Both* in vitro* and *in vivo* studies were subjected to this investigation. This review authenticates that rosemary has appeared as a worthy source for curing inflammation, analgesic, anti-anxiety, and memory boosting. It also arranges new perception for further investigations on isolated constituents, especially carnosic acid, rosmarinic acid, and essential oil to find exquisite therapeutics and support drug discovery with fewer side effects to help people suffering from nervous system disorders.

## Introduction

Nervous system disorders include abnormalities in either function or structure of the central or peripheral nervous system (1). These illnesses might be the result of trauma, metabolic dysfunction, infection or genetic conditions. A large number of scientific studies and discoveries aim to reduce the impacts and frequency of neurological disorders, mental health, and drug abuse.

Herbal medicines and natural products were used in ancient therapies (2). During the last decades, researchers focused more on herbs in drug discovery because of their limited side effects and fewer complications (3). According to the improving demand, the medicinal and pharmacological studies have been increasing worldwide (4).

**Figure 1:**
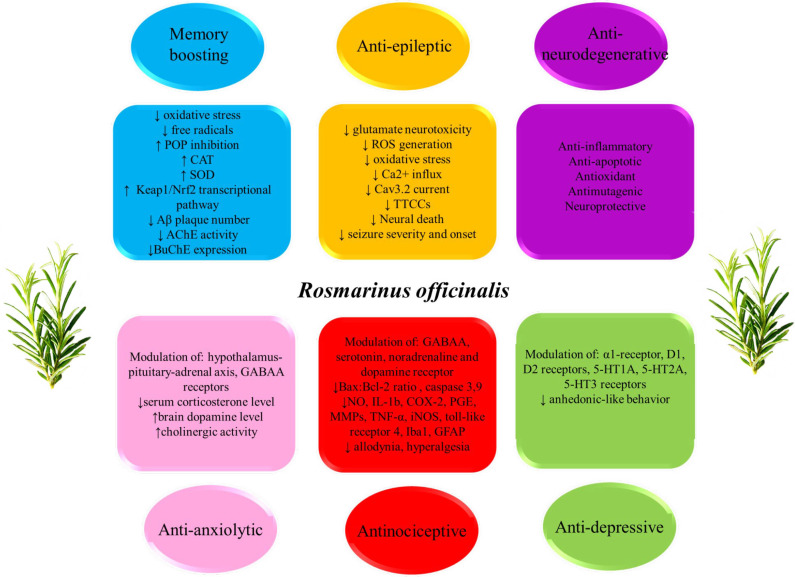
Neuropharmacological properties of rosemary on nervous system

Rosemary, *Rosmarinus officinalis* L. (Labiatae) has been used in folk medicine to alleviate several diseases including headache, dysmenorrhea, stomachache, epilepsy, rheumatic pain, spasms, nervous agitation, improvement of memory, hysteria, depression, as well as physical and mental fatigue (5, 6). Today, rosemary is grown worldwide but it is an evergreen perennial shrub native to southern Europe and Asia especially Mediterranean region (7). Recently, noticeable scientific interest is focused on the beneficial therapeutic properties of different kinds of rosemary extracts and its main constituents, such as carnosic acid, carnosol, rosmarinic acid, etc. A large number of studies either on animal models or cultured cells indicate the wide range medicinal properties of rosemary and its compounds such as anti-inflammatory (8, 9), anti-oxidant (10), antinociceptive (11), neuroprotective (12), antidepressant, anti-hysteric, ameliorative of memory and mental fatigue (13-15) (Figure 1). Moreover, the safety of rosemary has been displayed in various studies. The median lethal dose (LD_50_) value of methanolic extract of rosemary leaves prescribed intraperitoneally to mice was 4.125 g/kg of their body weight (16). Rosemary has also been classified as “generally safe” or GRAS (CFR182.10; 182.20) by the FDA in America (17). Rosmarinic acid was observed to have very scarce toxicity with an LD_50_ of 561 mg/kg in mice (18). The oral LD_50_ of carnosic acid was 7100 mg/kg in the acute toxicity in mice (19).

**Figure 2:**
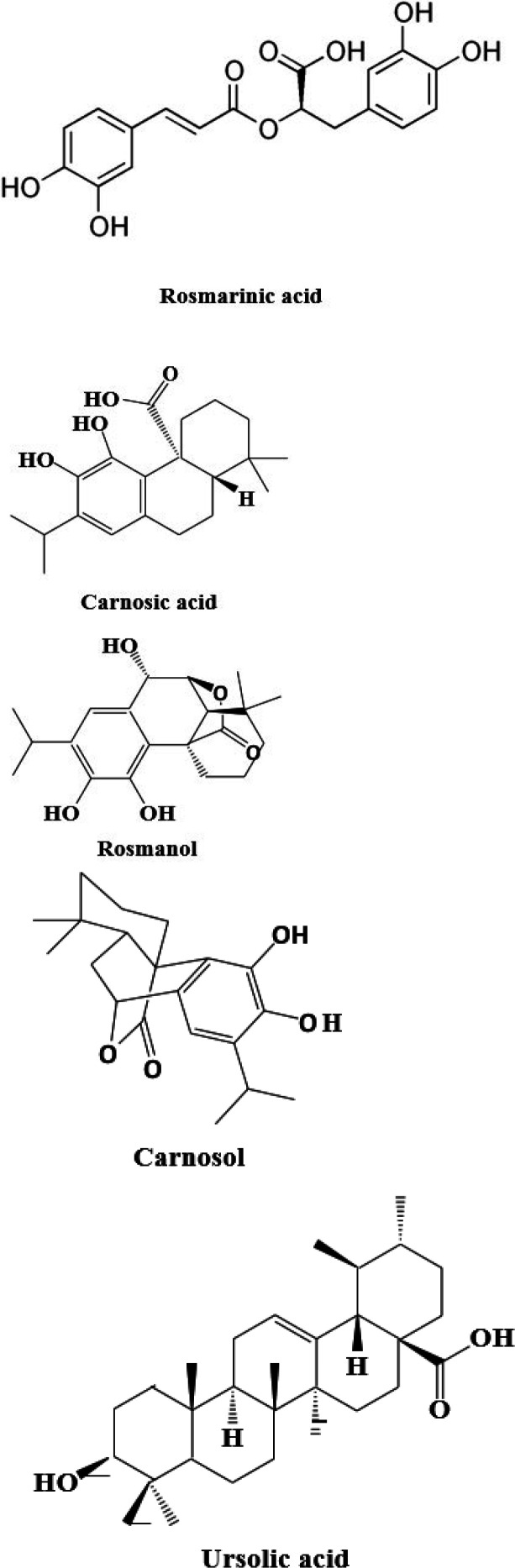
Chemical structures of some constituents of rosemary (*Rosmarinus officinalis* L.)

Phytochemical studies revealed that rosemary contains terpenoids, essential oils, alkaloids and flavonoids (20-22). Chemical analysis of different kinds of rosemary extracts composition reveals that the most potent active components are triterpenes, phenolic diterpenes and phenolic acids including rosmarinic acid, carnosic acid, rosmanol, carnosol, ursolic acid and betulinic acid (23, 24) (Figure 2). According to the documents, rosmarinic acid and carnosic acid possess the most medicinal effects among the mentioned phenolic compounds i.e. anti-inflammatory and anti-oxidants (25-28). Nowadays because of presence of many beneficial and un-useful constituents in medicinal plants it needs to focus on determination and effectiveness of the effective substances of extracts but not crude extracts.

## Methods

The data were gathered by searching the English articles in PubMed, Scopus, Google Scholar, and Web of Science. The keywords used as search terms were ‘*Rosmarinus officinalis*’, ‘rosemary’, ‘nervous system’, ‘depression’, ‘memory’, ‘Alzheimer’s disease’ ‘epilepsy’, ‘addiction’, and ‘neuropathic pain’. All kinds of related articles, abstracts and books were included. No time limitation was considered in this review. Both *in vitro* and *in vivo* studies were subjected to this investigation.

***Therapeutic effects of rosemary constituents on nervous system disorders***

*Depression*

Depression is a serious chronic psychiatric disease (29). Clinical and experimental studies have suggested several alterations occurred in neuronal noradrenergic and serotonergic function in the central nervous system (30). Another hypothesis focuses on the role of brain-derived neurotrophic factor (BDNF) in the brain (31). In addition, other studies point to the involvement of endogenous metabolites or inflammatory cytokines in the induction of depression (32).

The antidepressant-like effect of hydro-alcoholic extract of the leaves and stems of rosemary (100 mg/kg, PO) for 14 days was revealed in behavioral tests in mice and it was also shown that its antidepressant-like effect is dependent on its interaction with the noradrenergic (α 1-receptor), dopaminergic (D1 and D2 receptors) and serotonergic (5-HT1A, 5-HT2A and 5-HT3 receptors) systems (14). This research group also reported that chronic administration of the hydro-alcoholic extract of rosemary (10-300 mg/kg, PO) for 14 days similar to fluoxetine (10mg/kg, PO) could reduce anhedonic-like behavior and hyperactivity that were associated with hippocampal acetylcholinesterase (AChE) activity in olfactory bulbectomized mice (33). Although, more studies are necessary to determine which isolated compounds are responsible for the antidepressant-like effects of this extract. In fact, this is a major problem of using crude extracts in medicine.

In the extrapyramidal system of the brain, dopamine is a precursor to norepinephrine and epinephrine and it has an important role in behavior regulation (32). So, regulating the amount of dopamine and dopaminergic pathways is an important goal in controlling depression. It was also revealed that ursolic acid, a pentacyclic triterpenoid derived from rosemary could reduce the immobility time both in tail suspension test and forced swimming test in mice. Pretreatment with SCH23390 (0.05 mg/kg, SC, a dopamine D(1) receptor antagonist) and sulpiride (50 mg/kg, IP, a dopamine D(2) receptor antagonist) prevented the ursolic acid effects (0.001–10 mg/kg, PO) in the tail suspension test. Moreover, administrating the sub-effective dose of ursolic acid in addition to the sub-effective doses of SKF38393 (0.1 mg/kg, SC, a dopamine D(1) receptor agonist), apomorphine (0.5 μg/kg, IP, a preferential dopamine D(2) receptor agonist) or bupropion (1 mg/kg, IP, a dual dopamine/ noradrenaline reuptake inhibitor) decreased the immobility time in the tail suspension test compared with each of the drugs alone. These results show that the antidepressant effect of ursolic acid in the tail suspension test could be because of an interaction with the dopaminergic pathway and through activation of dopamine D_1_ and D_2_ receptors (34). This team also assessed the antidepressant-like property of different fractions of rosemary including, hexane (0.1-10 mg/kg, PO), ethanolic, ethyl acetate 1 and 2, and essential oil-free (0.1-100 mg/kg, PO), and some isolated compounds such as betulinic acid (10 mg/kg, PO), and carnosol (0.01-0.1 mg/kg, PO) in the tail suspension, a predictive test to investigate the antidepressant activity, in mice. Results showed that all of the fractions and prescribed constituents produced a significant antidepressant-like effect (35). This finding could be further evaluated by molecular and biochemical tests to determine the exact mechanisms involved in their antidepressant like properties.

By these documents, it may be suggested that antidepressant-like effect of rosemary could be, at least in part, because of carnosol, ursolic acid, betulinic acid and 1,8-cineole, the main compound in the essential oil of rosemary. A few of these studies are represented in Table 1.

*Memory, learning, and Alzheimer’s disease*

The number of elderly adults, over 65 years old, worldwide is supposed to be doubled by the year 2030 and to help individuals stay in the workforce longer, the need to stay cognitively fit is improving. Thus, the development of natural interventions to slow or prevent cognitive decline naturally associated with aging is crucial. Herbal ingredients and nutrients have been studied as a probable solution to this developing concern. One of the important hallmarks of the aging process is oxidative damage (36). The neuronal dysfunction observed in disorders associated with aging such as Alzheimer’s disease is mainly thought to be from oxidative stress. Free radicals are responsible for oxidative stress and aging (37). Aging and related diseases reveal when endogenous anti-oxidants are not able to counter free radicals damage to cells and cellular molecules (38). So, plant extracts with anti-oxidant ingredients might be a great help. In this regard a study by Farr *et al*. 2016, investigated the effects of rosemary extract contained 60% or 10% carnosic acid and spearmint extract contained 5% rosmarinic acid, anti-oxidant-based components of rosemary for 90 days, on memory and learning in mice and their results showed the positive effects of these ingredients on memory improvement in a mouse model (39).

It is known that inhibition of prolyl oligopeptidase (POP) might be effective in memory-related function (40). Rosmarinic acid (1, 2, 4, or 8 mg/kg, PO) for acute (4 training days) or 2 or 3 weeks sub-chronic periods inhibited POP activity and therefore showed a cognitive- improving effect in mice (41). These cognitive-enhancing effects of rosmarinic acid might be beneficial to populations of advanced age.

Song and colleagues, 2016, also confirmed the effect of rosemary extract containing 20% carnosic acid on the improvement of cognitive deficits in rats and it might be mediated by anti-oxidative (decreased ROS and increased superoxide dismutase (SOD)) and anti-inflammatory (reduced protein level of TNF-α, IL-6, and IL-1β in hippocampus) properties of rosemary (42). However, the pharmacological mechanisms behind the improvement in cognitive deficits are not clear enough and further examinations are needed to find the exact relationship between different doses of rosemary extract and improvement in cognitive deficits.

The inhalation of rosemary oil in 144 healthy volunteers induced subjective effects on mood as well as objective effects on cognitive performance (43). In another study, the aroma of rosemary oil improved performance in exam students by enhancing free radical scavenging activity and decreasing cortisol levels (44). In a study by Pengelly *et al*. 2012, rosemary powder (750 mg), the dose nearest to the normal culinary consumption, showed positive influences on the speed of memory, the time taken to effectively regain information from both episodic and working memory, on 28 older adults (mean age, 75 years) which is a useful predictor of cognitive function during aging (45). These results point to the value of further studies on the effects of different doses of rosemary on memory and cognition over the longer period of time.

Hippocampus is a part of the brain which has an important role in learning and memory, mood regulation, cognition and response to stress (46). It is one of the most vulnerable brain parts to oxidative stress (47). There are plenty of enzymatic and non-enzymatic anti-oxidant defense systems in cells to protect them from damages of free radical reactions (48). Since the endogenous anti-oxidant protection systems are not 100% effective, we assume that nutritional anti-oxidants could have beneﬁcial effects on the memory, neurogenesis, and activities of enzymatic oxidative in the brain. Rasoolijazi and colleagues, 2015, evaluated the effect of rosemary extract on memory and anti-oxidant status of the hippocampus in middle-aged rats. They reported that prescription of rosemary extract (50,100 and 200 mg/kg/day, containing 40% carnosic acid, o.p.) for 12 weeks in middle-aged rats increased spatial memory and the activity of SOD and chloramphenicol acetyltransferase (CAT) anti-oxidant enzymes (49).

Alzheimer’s disease is a complicated disease which implicates interaction between genetic and environmental risk factors and it is characterized by tau tangles, amyloid plaques, loss of synapses and neuronal loss (50). The generation of nitrosative and oxidative stress partially damage neurons, because oligomeric amyloid-β (Aβ) peptide triggers generation of reactive oxygen/nitrogen species (ROS/RNS) (51, 52). Activation of the Kelch-like ECH-associated protein 1-nuclear factor (erythroid-derived 2)-like2 (Keap1/Nrf2) pathway increases the transcription of anti-inflammatory proteins and phase 2 anti-oxidant. Hence, it could be a promising therapeutic process in various neurodegenerative conditions. It has been shown that carnosic acid converts to its active form by oxidative stress and its active form stimulates the Keap1/Nrf2 transcriptional pathway and therefore, produces phase 2 anti-oxidant enzymes in both *in vitro* and *in vivo* models (53, 54). In another research, the protective effects of carnosic acid were studied on primary neurons exposed to oligomeric Aβ in both *in vitro* and *in vivo* models. The histological results revealed that carnosic acid (10 mg/kg b.w., trans-nasally) expanded synaptic and dendritic markers, and decreased Aβ plaque number, astrogliosis, and phospho-tau staining in the hippocampus (55) (Table 1). Since carnosic acid is on the ‘generally regarded as safe’ (GRAS) list of FDA, same studies on the human for clinical approach will be useful.

It is also believed that prolong the existence of acetylcholine into the synaptic cleft might cause cholinergic function in Alzheimer’s disease due to inhibition of acetylcholine hydrolysis (56). It is proposed that cholinergic neurons degenerate in the basal forebrain and it is associated with loss of cholinergic neurotransmission in the cerebral part of the cortex. This might be therapeutically important because the cholinergic system of basal forebrain is involved in the attention and cognitive processing of memory (57). There are two major forms of cholinesterases in the human brain: butyrylcholinesterase (BuChE) and acethylcholinesterase (AChE). In the human brain, both of them are found in neurons, oligodendrocytes, astrocytes, tangles in Alzheimer’s disease and neuritic plaques (58). It is reported that AChE activity reduced in the cortex but BuChE activity increased or remained unchanged during Alzheimer’s disease development (59). A group of researchers assessed the influence of sub- chronic administration of rosemary extract (200 mg/kg, PO) on cognitive activities and behavior of rats and to evaluated BuChE and AChE gene expression level and activity in frontal cortex and hippocampus. It was observed that rosemary extract alleviated long-term memory and inhibited the AChE activity. It also had a stimulatory effect on BuChE in both parts of the rat brain. In addition, it reduced BuChE expression in cortex and increased it in the hippocampus (60). By the data in hand, it could be concluded that rosemary extract could improve long-term memory by inhibiting AChE activity in rat brain.

In order to check the possible effects of stimulation through the sense of smell on cognitive function, another team applied aromatherapy treatment on Alzheimer patients and proposed that aromatherapy might improve cognitive function, especially in Alzheimer patients (61).

*Epilepsy*

Epilepsy is a neurological disease that causes periodic spontaneous seizures and memory and learning deficits (62). Seizures lead to neuronal death because of over- activating of glutamate receptors (63). Glutamate has an important role in cognitive actions including learning and memory and in synaptic plasticity as well, but the higher concentration of glutamate and over activation of its receptor leads to neurodegeneration in the central nervous system (64). According to previous studies, glutamate neurotoxicity is because of generating ROS damages to cellular organelles like mitochondria (65). Thus, substances which are able to neutralize ROS could protect neurons and prevent subsequent death. Anti-oxidant components of rosemary extract (250, 500 and 750 mg/kg) reduced lipid peroxidation and interact with the free radical chain reaction and donate hydrogen and finally neutralize harmful agents in cooking liver pâté (66). Another study showed that rosemary extract (100 mg/kg/day, containing 40% carnosic acid, PO, for 23 days) might improve working and spatial memory deficits and neuronal degeneration induced by the toxicity of kainic acid (9.5 mg/kg, IP) in the hippocampus of rats, which might be because of its anti-oxidant properties. Rosemary also significantly decreased both seizure severity and onset in rats. In addition, neuronal loss in the CA1 region reduced (67). Although, the mechanisms of this improving effects of rosemary has not been well understood and need to be further investigated.

Previous studies reported that oxidative stress increased Ca^2+^ influx from extracellular fluid into neurons (68, 69). Ca^2+^ driven by the endoplasmic reticulum might raise Ca^2+^ concentration as well. The increased Ca^2+^ concentration raises amount of Ca^2+^ in nuclei and mitochondria and finally leads to disrupting normal metabolism and neurodegeneration. T-type calcium channels (TTCCs) play important roles in neuroprotection, neuronal excitability, sleep, and sensory processes. They are also involved in pain and epilepsy. Diversity in the functional properties of T-type calcium channels is further supported by molecular investigations that have explained three genes encoding these channels: CaV3.1, CaV3.2, and CaV3.3 subunits (70, 71). In a research done by El Alaoui, 2017, essential oil and methanolic extract of rosemary, as well as rosmarinic acid, inhibit the Cav3.2 current in a concentration-dependent manner in HEK-293T cells. Furthermore, they induce a negative shift of the steady-state inactivation of CaV3.2 current with no change in the activation properties. These results suggest that the inhibition of TTCCs might contribute to the neuroprotective and anxiolytic effects of rosemary (72). Taken together, these findings support a pharmacological modulation of TTCCs by rosemary and suggest that TTCC inhibition might contribute to the anticonvulsant and neuroprotective properties of this medicinal plant. TTCCs might therefore, represent a novel molecular target for rosmarinic acid; although further studies are needed to investigate the efficacy of rosmarinic acid to possibly regulate other ion channels. Some performed studies on addiction are summarized in Table 2.

*Addiction*

Using opiates is a global epidemic and it continues to spread. Finding a non-addicting agent to prevent the addiction process is one of the main concerns of researchers in this field, however, it has not yet fully solved (73). Opioid withdrawal symptoms include nausea or vomiting, rhinorrhea, dysphoric mood, muscle aches, pupil dilation, lacrimation, sweating, piloerection, yawning, diarrhea, insomnia and fever (74). Previous studies conducted on lab animals have reported that *R. officinalis* could be effective in reducing symptoms of opioid withdrawal syndrome. It has been observed that analgesic properties of alcoholic (0.96 g/kg, IP, for 4 days) and aqueous (1.68 g/kg and 2.4 g/kg, IP, for 4 days) extracts of rosemary have been antagonized by naloxone (5 mg/kg, SC). Thus, it might reinforce the interaction of rosemary with opioid receptors (22, 75) (Table 2). In another study, in 81 patients, it has been confirmed that rosemary (8-16 capsules/day, containing 300 mg dried leaves of rosemary) could be used as an herbal medicine for alleviating withdrawal syndrome symptoms during treatment strategies for opium addiction and likely addiction to other opioids. In this study, the effectiveness of rosemary in the reduction of insomnia, musculoskeletal pain in opium addicts and improvement of sleep was clearly demonstrated during 4 weeks (76). It is probable that the anticonvulsant effects observed in the former studies occur with the same mechanisms as rosemary’s effects on reducing insomnia in this study. An investigation by Hosseinzadeh *et al*. 2006, showed that rosemary can decrease muscle jerks produced by morphine withdrawal syndrome (75). These beneficial properties of the plant might be attributed to psycho-stimulant and anti-inflammatory effects (8, 77). These documents revealed that rosemary might be used as an elective complementary compound to modify withdrawal syndrome through treatment procedure for opium addiction and likely addiction to other opioids.

*Neuropathic pain*

Neuropathic pain is known as pain caused by a disease or lesion of the central or peripheral nervous system by features like hyperalgesia and allodynia (78). Recently, it has been reported that pro-inflammatory cytokines including interleukin-1b (IL-1b) produced by immune cells, microglia, and astroglia, in the spinal cord have important roles in the pathogenesis of neuropathic pain (79). These agents can initiate a cascade of neuro-inflammation-related events that might keep up and worsen the original injury that finally leads to pain and chronicity (80). Moreover, inflammation induces cyclooxygenase-2 (COX-2) expression and results in the generation of prostaglandins (PGE) (81). PGE2 is a pain-inducing factor. It is able to sensitize primary sensory neurons and leads to central sensitization and also facilitate the release of pain-related neuropeptides (82). Metalloproteinases (MMPs) are mostly involved in tissue remodeling and inflammation associated with some neurodegenerative disorders (83). These agents have important roles in nociception and hyperalgesia in the chronic phase of neuropathic pain (84). Studies in these fields demonstrated that hydroalcoholic extract of rosemary (10-50 mg/kg, IP) and carnosol (0.5-2 mg/kg, IP) inhibit formalin-induced pain and inflammation in mice (88). In a previous study, it was reported that different triterpenes (micromeric, oleanolic, and ursolic acids) in *R. officinalis* revealed anti-inflammatory and antinociceptive properties in experimental models of pain including acetic acid-induced writhing test, formalin test, and a model of arthritic pain in mice. Moreover, each of the mentioned triterpenes revealed a similar capability to that observed with ketorolac (10 mg/kg, IP), a non-steroidal anti-inflammatory medicine and a typical clinic analgesic (86). González-Trujano and colleagues, 2007, studied the antinociceptive effect of ethanol extract of rosemary aerial parts. They compared the antinociceptive property of this herb with either tramadol (3.16-50 mg/kg, IP in mice, and 1.0-31.62 mg/kg, IP in rats) or acetylsalicylic acid (31.62-562.32 mg/kg, PO). The achieved data indicate that aerial parts of rosemary have antinociceptive and anti-inflammatory properties, and consolidate the use of it in folk medicine (11). In this regard, Ghasemzadeh *et al*. 2016, conducted a research to investigate the potential anti-inflammatory properties of ethanolic extract of *R. officinalis* (100, 200, and 400 mg/kg, IP) and rosmarinic acid (10, 20, and 40 mg/kg, IP) in a rat model of sciatic nerve chronic constriction injury (CCI)-induced neuropathic pain. In this study, the effects of 14 days, intraperitoneal prescription of ethanolic extract of rosemary and rosmarinic acid on the lumbar spinal cord expression of oxidative stress and inflammatory markers including PGE-2, IL-1b, COX2, NO, and MMP2 were assessed (87). Histological analysis of the sciatic nerve revealed that terpenoid-enriched rosemary extract prevented axon and myelin derangement, edema, and inflammatory infiltrate (88). The obtained data reinforced the traditional use of rosemary as an effective treatment for inflammatory disorders and pain relief. These data also suggest that the ethanolic extract of rosemary and rosmarinic acid might be potential candidates in treating neurological disorders accompanied by inflammation and neuropathic pain by modulating neuro-inflammation. According to the data, it could be suggested that the extract and rosmarinic acid might have an important role against oxidative and inflammatory markers including IL-1b, PGE-2, NO, COX-2, and MMP2.

As previous studies reported, the apoptosis process is activated in the dorsal horn of spinal cord after CCI surgery of sciatic nerve (89). However, the relation between neuronal apoptosis in the spinal cord and the occurrence of hyperalgesia and allodynia is not fully known yet. Apoptosis may cause structural changes in neurons, increase the sensitivity of the nociceptive system and finally induce hyperalgesia or allodynia (90). Astrocytes and microglia might have regulatory roles in neuropathic pain by releasing chemokines and cytokines. Microglia and astrocytes have different neuronal activity. However, sometimes their activities overlap in mediating CNS innate immune responses. Both of these cells are activated following nerve injury and might lead to inflammatory reactions and pathological impacts such as neuronal chronic inflammation, toxicity, and hyper-excitability (91). Thus, it could be concluded that anti-inflammatory and anti- apoptotic reactions may lead to the anti-hyperalgesic and anti-allodynic effects of rosemary after nerve injury. Some other research projects have been designed to investigate the underlying mechanisms of the alcoholic extract of rosemary and one of its main constituents, rosmarinic acid on neuropathic pain on rats. The results suggest that alcoholic extract of rosemary (100, 200, and 400 mg/kg, IP) and rosmarinic acid (10, 20, and 40 mg/kg, IP) reduced inflammatory responses by decreasing apoptosis-related mediators (Bax, cleaved caspase- 3, and 9), inflammatory factors (TNF-α, iNOS, toll-like receptor 4) and the protein levels of glial activation markers (Iba1, GFAP) in rats’ spinal cords. Rosmarinic acid might be partially responsible for observed protective effects (92, 93). These studies might offer a new potent and promising therapy in alleviating neuropathic pain, however, more research into the antinociceptive mechanisms of rosemary and its components as well as clinical studies on patients suffering from chronic conditions of pain will be mandatory.

In another study analgesic effects of rosemary essential oil (10, 20 mg/kg, PO) and its pharmacodynamics interactions with paracetamol (acetaminophen) (60 mg/kg, IP) and codeine (30 mg/kg, IP) were investigated in mice. Their results support the use of rosemary in pain management and show a therapeutic potential of rosemary essential oil in combination with analgesic medicines (94). In line with this study, the data of another research showed that rosemary essential oil (70, 125, 250 mg/kg) had a significant antinociceptive influence in the acetic acid-induced abdominal writhing test (95). By the achieved data, it might be concluded that rosemary essential oil has anti-inflammatory and peripheral antinociceptive activity. A research examined the effect of the rosemary essential oil on analgesic effect and percutaneous absorption of diclofenac topical gel in mice and it was observed that rosemary essential oil (0.1, 0.5, and 1.0% w/w) enhanced diclofenac percutaneous absorption (96). 

Abdelhalim and colleagues, 2015, studied the effects of non-volatile constituents of rosemary including cirsimaritin, rosmanol, and salvigenin (50-200 mg/kg) on the central nervous system function. These components show biphasic modulation of GABAA receptors and demonstrated CNS activity in mouse models of antinociception (97) (Table 3). But, further studies have to be done to find the probable antinociceptive mechanisms of these substances and to investigate the effect of these compounds on GABAA receptor subtypes.

Previous studies reported that 54% of hemodialysis patients suffer from pain (98). Furthermore, 64% of pain is due to musculoskeletal issues and reveals in the legs (99). The experience of chronic pain has negative effects on patients; the resulting immobility causes high prevalence of depression, irritability, inability to cope with stress, increased fatigue, and reduced the quality of life (100). The topical application of rosemary was able to alleviate the frequency and severity of recurrence of musculoskeletal pain in these patients (101). So, rosemary induces its analgesic properties by affecting different antinociceptive pathways. But further detailed investigations are essential to determine the exact involved mechanisms through which rosemary exhibits its antinociceptive activities such as the number of inflammatory cells, apoptotic and microglial activation markers or the probable direct effect of rosemary on muscles.

Diabetes mellitus could also cause neuronal tissue damage in the central and peripheral nervous system. A study have reported that more than one-half of diabetic patients suffer from diabetic neuropathy and pain due to diabetes neuropathy (102). In diabetic patients, hyperglycemia is reported to be the main underlying factor of injury to the nervous system (103). Some previous results have led to the proposal that diabetic neuropathy might occur because of constant production of reactive oxygen species through glucose auto-oxidation and development of glycation end-products, activation of nuclear enzyme Poly(ADP-ribose) polymerase (PARP), and reduction of anti-oxidant protection (104). Moreover, apoptosis has been reported to be another probable mechanism for the high glucose-induced neural disorder and cell death (105). The neuroprotective and anti-hyperalgesic effects of rosemary extract (100, 150, or 200 mg/kg, PO) in a rat model of streptozotocin-induced diabetes were studied for 21 days. It was observed that treating with rosemary extract improved hyperalgesia, hyperglycemia, and motor deficit, decreased caspase-3 activation and Bax: Bcl-2 ratio (106). In another study it was also concluded that different rosemary extracts and its main phenolic components exert advantageous properties against diabetes and metabolic syndrome through increasing insulin secretion and response, inhibition of advanced glycation end products generation, suppression of gluconeogenesis, anti-oxidant, anti-inflammatory, and anti- hyperlipidemic properties. These magnificent effects are systematically related to enzyme modulation, transcription factors, various vital signal transduction pathways, and important gene expressions (107). Although there are several documents which examined the neuroprotective and analgesic effects of rosemary extracts in animal studies and *in vitro* investigations, more clinical assessments are essential to support the safety and potency of the phenolic agents of rosemary in humans. Hence, it can be finalized that rosemary extract has anti-hyperalgesic and neuroprotective properties in diabetes.

Generally, pain has a negative impact on quality of life. Considering the limited effectiveness of current medications, it is necessary to study the effects of different complementary therapies such as aromatherapy massage (Swedish massage therapy using massage herbal essential oils). After inhaling essential oil molecules or their absorbance through the skin, these molecules stimulate hippocampus and amygdala and initiate their impact on emotional, physical and mental health (108). The antinociceptive effects of aromatherapy could be related to the following mechanisms: 1. the complex mixture of volatile chemical agents might reach pleasure memory sites in the brain; 2. certain analgesic factors within essential oils that may affect some neurotransmitters including serotonin, noradrenaline and dopamine receptor sites in the brain; 3. the interaction of touch sense with sensory neurons in the skin; and 4. increased rate of essential oil absorption into the bloodstream (109). In this field, a study aimed to investigate the effects of rosemary essential oil in aromatherapy massage on the quality of life and severity of neuropathic pain in 46 patients with diabetes. They reported a reduction in scores of neuropathic pain significantly and an increase in scores of quality of life (110). Thus, essential oil of rosemary could be safely used in a clinical setting by nurses. However, experience and training are critical to gain positive results.

*Stress and anxiety*

Emotional disorders, such as anxiety, cause a huge burden on health all around the world. Documents suggest that stress might lead to the loss of neuronal cells, atrophy and reduce the volume of key structures in the brain. Long-term exposure to stress may induce neuronal degeneration, neuronal inflammation and brain microdamage (111). Chronic stress occurs because of high glucocorticoids level and hyperactivity of hypothalamus- pituitary-adrenal axis that triggers several physiological adaptive feedback regulatory mechanisms (112). Furthermore, some documents have displayed that stress stimulates ACh release in a brain region-specific method (113, 114). In previous research projects it has been claimed that *R. officinalis* contains polyphenols such as rosmarinic acid, luteolin, carnosic acid, and other components that possess several effects on psychiatric disorders or neurological functions such as anti- depressive and anti-anxiety properties and neuroprotective and cognitive effects (15,115,116). It has also been reported that rosemary essential oil exhibited improved mood and cognition in healthy adults (43). The inhalation of rosemary essential oil as an anti-stress (117, 118) and anxiolytic (117-121) therapy has fewer side effects (122). The detailed mechanisms and effectiveness of this essential oil on neurological and psychological function is not well-understood. In a recent study, the effect of inhalation of rosemary essential oil was evaluated on the molecular mechanism that reduces stress *in vitro* using PC12 cells and *in vivo* using mice. The results showed that inhalation of rosemary essential oil decreased stress by reducing serum corticosterone level and increased brain dopamine level *in vivo*. Thus, this essential oil might modulate the activities of the sympathetic nervous system and hypothalamic–pituitary–adrenal (HPA) axis. In addition, it has been reported that rosemary essential oil regulates brain neurotransmitter activity and demonstrates neurophysiological effect related to acetylcholine synthesis and release, as well as inducing neuronal differentiation in mice (123). Moreover, *R. officinalis *L. has been claimed to activate cholinergic activity (AChE activity) in PC12 cells via phosphorylation of ERK1/2 (124). More investigations might be useful to examine the validity of these results in clinical trials. The results also reinforce that rosemary essential oil has potential properties to be used as a safe alternative treatment method for stress-related mood disorders.

Liquid chromatography-mass spectrometry analysis of rosemary tea showed the presence of 16 compounds that are classified into the categories of flavonoids, diterpenes, and hydroxycinnamic derivatives; it was shown that rosmarinic acid was the major bioactive compound of the infusion, followed by a caffeic acid derivative and luteolin 7-O-glucuronide. Rosmarinic acid was also the major component of the water-soluble extract of rosemary leaf in the study of del Bano *et al*. 2003, but it was found only in trace amounts in methanol and acetone extracts (125). Rosemary tea (2% w/w) prescription employs anxiolytic and antidepressant properties on mice and inhibits ChE activity; its main phytochemicals might affect in a similar way as inhibitors (126) (Table 3). An *in vivo* study showed that when rosmarinic acid was intraperitoneally administered to adult male mice, it reduced significantly the immobility time during the forced swim test (127). Caffeic acid also demonstrated antidepressant effects. Moreover, a dose-dependent anxiolytic action of rosmarinic acid (1, 2, 4 or 8 mg/kg) was observed when it was administrated intraperitoneally to adult male mice (128). Rosemary (500 mg, twice daily, for a month) as a traditional herb might be used to enhance prospective and retrospective memory, reduce anxiety, depression, and promote sleep quality in university students (129). Herbal extracts are chemically complex mixtures containing several compounds with multiple potential targets and mechanisms. Hence, more investigations are necessary to explain the involved mechanisms, although the behavioral effects have been definitely demonstrated in several studies.

*Parkinson’s disease*

Parkinson’s disease (PD) is a neurodegenerative illness that is caused by a loss of dopaminergic neurons in the substantia nigra. The clinical symptoms of PD are characterized by a combination of bradykinesia, resting tremor, rigidity, and postural instability (130). The brain in PD is more susceptible to oxidative damage because it is rich in polyunsaturated fatty acids and has high oxygen utilization. Recent studies have suggested that oxidative stress is implicated in the dopaminergic neuronal cell death in PD (131). In rotenone-induced neurotoxicity of cultured dopaminergic cells carnosol significantly increased the amount of tyrosine hydroxylase, an enzyme that is down-regulated in Parkinson’s disease (132). Carnosic acid protected against 6-hydroxydopamine-induced neurotoxicity in a rat model of Parkinson’s disease which is probably attributable to its anti-oxidative and anti- apoptotic properties. The present data might help to distinguish the possible mechanisms of rosemary in the neuroprotection of PD (133). Carnosic acid had the potential for neuroprotection both *in vivo* and *in vitro*. Carnosic acid protected against 6-hydroxydopamine-induced neurotoxicity by inducing anti-oxidant enzymes and inhibiting cell apoptosis. So, carnosic acid could be a potent candidate for protection against neurodegeneration in PD.

**Table 1:** Clinical studies of rosemary and the active constituents on depression, memory and learning

Subjects	Type of extract, constituents/ doses/ Time of exposure	Endpoints	Reference
Mice	Hydroalcoholic extract; 10–300 mg/kg; 14 days	- The hydroalcoholic extract drove back the olfactory bulbectomy -induced hyperactivity, amplified exploratory and anhedonic behavior. - It improved serum glucose level and decreased hippocampal AChE activity in bulbectomized mice.	(30)
Mice	Ursolic acid; 0.01 and 0.1 mg/kg,	- Ursolic acid decreased the immobility period in the tail suspension test (0.01 and 0.1 mg/kg) and also in the forced swimming test (10 mg/kg). - The effect of ursolic acid (0.1 mg/kg) in the tail suspension test was prevented by the pretreatment of SCH23390 (0.05 mg/kg, a dopamine D 1 receptor antagonist) and sulpiride (50 mg/kg, a dopamine D 2 receptor antagonist).	(31)
Wistar rats	Extract (containing 40% carnosic acid); (50,100 and 200 mg/kg/day); 12 weeks	- The extract (100 mg/kg) recovered the spatial memory retrieval score. - SOD, GPx and CAT enzymes significantly elevated in comparison with the normal group.	(46)
(hAPP)-J20 mice and (3xTg AD) mice	Carnosic acid; 3 months	- Carnosic acid treatment of hAPP-J20 mice alleviated memory and learning in the Morris water maze test. - Carnosic acid increased dendritic and synaptic markers, and reduced astrogliosis, Aβ plaque number, and phospho-tau staining in the hippocampus.	(52)

**Table 2:** Clinical studies of rosemary and the active constituents on epilepsy and addiction

Subjects	Type of extract, constituents/ doses/ Time of exposure	Endpoints	Reference
Rats	Extract (containing 40% carnosic acid); 100 mg/kg; 23 days	- Neuronal loss in CA1 decreased remarkably in the animals in Kainic Acid (9.5 mg/kg) + extract group. - Spatial memory impairment reduced in the animals in Kainic Acid (9.5 mg/kg) + extract group. - Shuttle box test showed that passive avoidance learning disability obviously, boosted in the animals in the mentioned group.	(64)
HEK-293T cells	Methanolic and essential oil extracts	- Both the methanolic extract and essential oil of rosemary inhibit Cav3.2 current in a concentration-dependent manner. - These extracts compel a negative shift of the balanced inactivation of CaV3.2 current with no alteration in the activation properties.	(69)
Mice	Aqueous and ethanol extracts; (1.68, 2.4 g/kg) and (0.96 g/kg) respectively; 4 days	- Both extracts lessened the number of jumps after naloxone injection.	(18)
Mice	Aqueous, methanolic-aqueous and chlorformic fractions; (0.96 g/kg and 1.68 g/kg), 4 days	- All fractions reduced the number of jumps when they were injected 1 h before the last dose of morphine.	(72)

**Table 3:** Clinical studies of rosemary and the active constituents on neuropathic pain, stress and anxiety

Subjects	Type of extract, constituents/ doses/ Time of exposure	Endpoints	Reference
Rats	Alcoholic extract; (100, 200, and 400mg/kg); 14 days	- All three mentioned doses of rosemary extract reduced neuropathic behavioral changes as compared with CCI animals that received the vehicle. - Rosemary extract, 400mg/kg notably declined the levels of Bax, cleaved caspases 3 and 9, Iba1, TNF-α, iNOS and TLR4 in comparison with vehicle-treated CCI animals.	(84)
Mice	Rosmanol, cirsimaritin and salvigenin; (50-200 mg/kg)	- They elicited anxiolytic, antinociceptive, and antidepressant properties. - These compounds were indicated to possess biphasic modulation of GABA_A_ receptors.	(95)
Mice	essential oil	- Inhalation of rosemary essential oil considerably minified the immobility time of mice and serum corticosterone level, accompanied by increased brain dopamine level.	(121)
Mice	Rosemary tea; (2% w/w); 4 weeks	- Cholinesterase isoforms activity was lowered in the brain of rosemary treated group.	(124)

## Conclusion

The present review demonstrates that the main ethnopharmacological uses (anti-spasm, analgesic, anti-inflammatory, anti-anxiety and memory-boosting) of rosemary have been validated by neuropharmacological investigations. By reviewing the previous literature, it is concluded that the most important components of rosemary which are medicinally and pharmacologically active are rosmarinic acid, carnosic acid, and the essential oil. These compounds can provide promising natural medicines in the treatment of the nervous system pathological conditions including anxiety, depression, Alzheimer’s disease, epilepsy, Parkinson’s disease, and withdrawal syndrome.

It is also noteworthy to mention that studies regarding herbal medicines should be taken into more consideration because the safety and efficacy of many herbal medicines are still unclear. Also, additional reliable trials are essential to evaluate the safety and efficacy of different constituents of rosemary in treating different nervous system disorders. Furthermore, the probable mechanisms of action and the potential antagonistic and synergistic properties of multi-component mixtures of rosemary need to be examined by the integration of physiological, pharmacological, bioavailability-centered, and pharmacokinetic methods. Prolonged and high dose usage of traditional formulations of rosemary and its active constituents should be avoided until more profound toxicity investigations become available. The new findings may expand the present therapeutic importance of rosemary and develop its future use in modern medicine. 
